# Once-weekly oral medication with alendronate does not  prevent migration of knee prostheses: A double-blind randomized RSA study

**DOI:** 10.1080/17453670902804968

**Published:** 2009-02-01

**Authors:** Ulrik Hansson, Sören Toksvig-Larsen, Leif Ryd, Per Aspenberg

**Affiliations:** ^1^Department of Orthopedics, Lund University HospitalLundSweden; ^2^Department of Orthopedics, Karolinska University HospitalHuddingeSweden; ^3^Department of Orthopedics, Linköping University HospitalLinköpingSweden

## Abstract

**Background and purpose** Early migration of joint replacements is an effect of poor fixation and can predict late loosening. By reducing the bone resorption after implantation of a joint replacement, it should be possible to enhance the initial fixation of the implant. We studied the effect of once-weekly treatment with alendronate after knee replacement.

**Patients and methods** We recruited 60 patients (60 knees) with gonarthrosis who were scheduled for a total knee replacement. They were operated on with identical implants and uncemented fixation. 30 patients were treated with a bisphosphonate (alendronate) and 30 patients underwent placebo treatment. The treatment started postoperatively and continued on a weekly basis for 6 months. The fixation of the implants was measured with repeated radiostereometry for 2 years.

**Results** There was no difference in migration of implants between the two groups.

**Conclusion** With uncemented fixation of knee implants, no benefit of once-weekly treatment with alendronate, starting postoperatively, could be seen during a 2-year follow-up period.

## Introduction

Early migration of total joint replacements can be predictive of late loosening (Freeman and Plante-Bordeneuve 1994, Kärrholm et al. 1994, Ryd et al. 1995, Stocks et al. 1995). This indicates that the risk of loosening is determined peroperatively (Ritter et al. 1999) or that crucial pathophysiological events occurring during the first postoperative months determine the risk of late loosening (Mjöberg et al. 1994, van der Vis et al. 1998a).

From radiostereometric (RSA) studies, it has been established that tibial components migrate postoperatively (Ryd et al. 1995, Ryd 1986, Nilsson et al. 1991, Hilding et al. 1995), mainly by sinking into the bone bed. Most implants have become settled by 6 months to 1 year, but some continue to migrate. A study of 143 knee implants followed longitudinally by RSA showed that all knees that were finally revised due to aseptic loosening had been migrating continuously since the primary implantation (Ryd et al. 1995). Furthermore, continuous migration is associated with inducible displacement, i.e. immediate motion between implant and bone upon loading (Hilding et al. 1995). Since the tibial component of a knee prosthesis, or the cement surrounding it, rests directly on bone postoperatively, inducible displacement suggests that the superficial layer of the bone has been resorbed and replaced by soft tissue (Levitz et al. 1995).

The tibial bone bed is likely to be necrotic after surgery, because of trauma and damaged microcirculation. Thus, the remodeling that ensues has similarities with fracture repair or bone modeling rather than with normal remodeling. The coupling between resorption and new bone formation, which is seen in normal remodeling, cannot be expected here. Thus, if the necrotic bone trabeculi are resorbed before new ones are produced, softening and collapse may occur. This may reduce the mechanical support of the prosthesis. If this scenario is correct, reducing resorption would be a more efficacious way of inhibiting or preventing loosening of implants than addressing the pressure in the effective joint space, i.e. the periprosthetic regions that are accessible to joint fluid or the wear particles generated in all joint replacements. Bisphosphonates are effective inhibitors of bone resorption (Wise et al. 2005). Enhancing effects of bisphosphonates on postoperative bone mineral density in distal femurs and proximal tibias after joint replacement have already been shown (Wang et al. 2006). The effect on fixation of implants, as measured by RSA, has been studied with cemented prostheses and a bisphosphonate (clodronate) given daily (Hilding et al. 2000). The aims of our study were to enhance the initial fixation also in uncemented prosthesis and to simplify the treatment by using alendronate administered on a weekly basis. The treatment period was from surgery to 6 months. The primary effect variable was the maximal total point motion, as measured by RSA after 2 years.

## Patients and methods

60 patients were recruited for this study, after performing power calculations (see below). Inclusion criteria were gonarthrosis stage 3–5 according to Ahlbäck (1968), and age 50–80 years. Exclusion criteria were cortisone or bisphosphonate medication, rheumatoid arthritis or any other systemic illness affecting the skeleton, or generally poor health. Patients were randomly allocated to the treatment (alendronate) group or a placebo group. The patients were randomized after surgery by opening a sealed envelope, when the operative procedure had ascertained that the case was suitable for uncemented fixation. Age and sex distribution were similar in the 2 groups (Table 1). The patients were informed before they were to be operated with total knee arthroplasty, and written consent was obtained. Merck, Sharp and Dohme provided the tablets, either alendonate (70 mg) or placebo. The unit catering for clinical trials at the pharmacy of Lund University Hospital packed and labeled the tablets, and neither the patients nor the investigators were aware of the coding.

**Table 1.** Demographic data

	Mean age	No. of patients	
	at surgery	at surgery	at follow-up
Alendronate	68 (51–80)	30 (17 F)	24 (14 F)
Placebo	69 (54–80)	30 (18 F)	21 (14 F)

2 surgeons performed the operations in a standardized manner with an uncemented, non-hydroxyapatite coated, porous-coated total knee prosthesis (Duracon; Stryker Inc.). Tantalum markers of 0.8 mm were implanted in the proximal tibia (8–10 markers) and in the plastic insert (4 markers). Radiographs for RSA were taken on the fifth to seventh postoperative day, and at 6 weeks, 3 and 6 months, and 1 and 2 years. 2 films were taken simultaneously at a 90° angle with the knee inside a calibration cage, according to standard RSA. The digitization of the radiographs and the calculations by the programs X-ray, Kindat, and winRSA were performed in Lund. The ethics coSORTtee of Lund University and the Swedish Medical Products Agency approved the protocol of the study.

On the day after the operation, the patients started the oral medication according to the routine for weekly osteoporosis treatment.

In order to secure patient compliance a nurse had regular telephone contact with the patients, to ensure that they remembered to take their tablets. The tablets were given to the patients on 3 occasions, the first when leaving hospital after surgery, the second at the 6-week follow-up, and the third after 3 months. After the treatment period, the patient compliance regarding the pharmacological treatment was calculated from the amount of tablets in the packages that were returned.

### Statistics

The power analysis was mainly based on another study dealing with the specific type of uncemented prosthesis to be used in this study. Prosthetic migration (as maximal total point motion, n = 14) was log-normally distributed with an SD of 0.20. A decrease of 30% meant a difference in log value of 0.15. This yielded a power of 83% to find a significant (p = 0.05) decrease in migration with 2 groups of 30 patients.

Results were analyzed according to the intention-to-treat principle (Hollis and Campbell 1999), so that all patients were considered to belong to the group to which they were originally randomized, regardless of whether they took take their tablets or not. Maximum total point motion (MTPM) at 2 years served as primary effect variable, but at all time points the results of segment motion with 6 degrees of freedom were also studied. Segment motion was analyzed using Student’s t-test. The primary effect variable was not normally distributed in both groups, and was tested by Mann-Whitney U-test.

## Results

In the alendronate group, 24 patients (14 females) remained after exclusion due to the onset of severe disease causing inability to fulfill treatment (2), RSA problems (3), or unwillingness to complete the study (1). The numbers for the placebo group were 21 (14 females) after exclusion of 9 patients (onset of severe disease (2), RSA problems (3), lost or unwillingness to complete the study (4).

**Figure 1.**
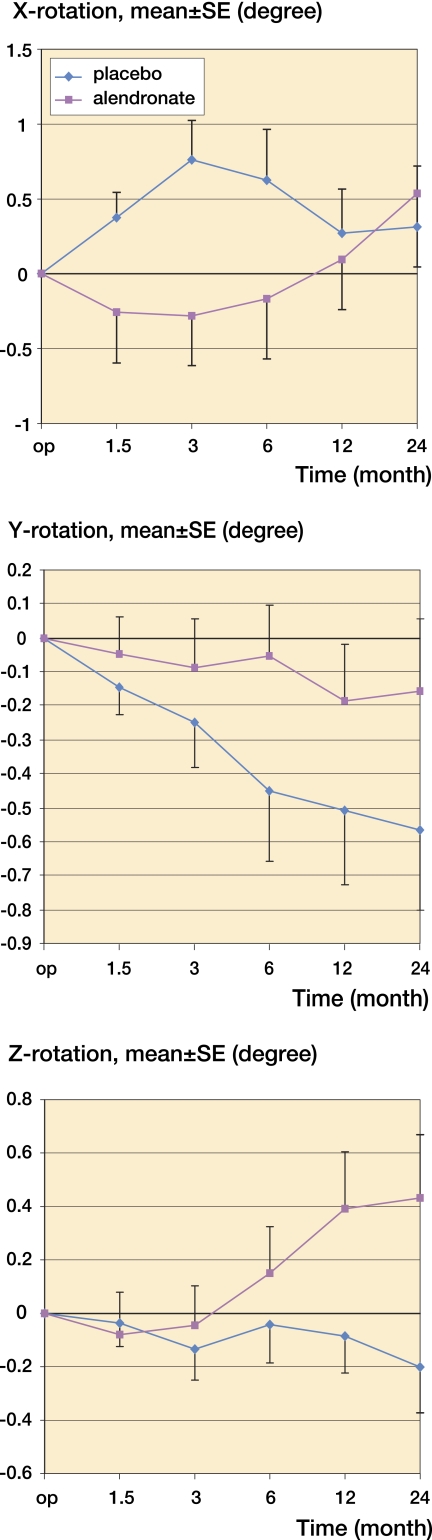
Rotation of the tibial components in 3 directions during the observation period.

**Figure 2.**
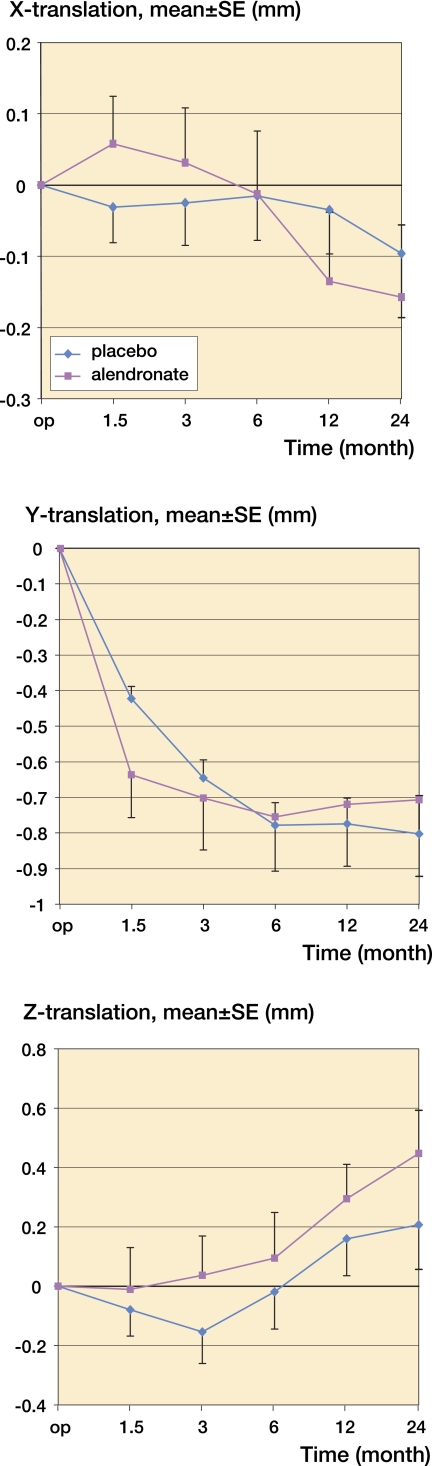
Translation of the tibial component in 3 directions during the observation period.

**Figure 3.**
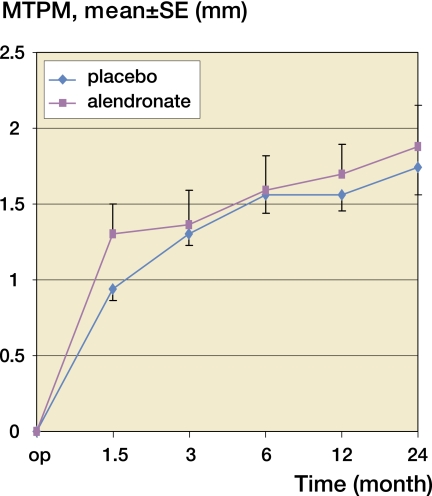
The movement of the single marker that moves the most, maximal total point motion (MTPM).

Patient compliance regarding the pharmacological treatment was 90% in both groups. 2 patients in each group took less than 75% of the tablets. Both groups had similar RSA findings and no statistically significant difference was found (Figures 1–3). We found no significant differences regarding maximal total point motion (MTPM) at the 1- and 2-year follow-up RSA examinations (Table 2).

**Table 2.** Description of the results regarding RSA analysis of the migration of the marker that moved the most

MTPM		n	Mean	SD	SE	95% CI	Range
1 year	placebo	20	1.57	0.53	0.12	1.32–1.82	0.63–2.43
	alendronate	23	1.69	0.97	0.20	1.28–2.11	0.42–4.77
	total	43	1.63	0.79	0.12	1.39–1.88	0.42–4.77
2 years	placebo	19	1.74	0.81	0.19	1.35–2.13	0.66–3.73
	alendronate	22	1.89	1.25	0.27	1.33–2.44	0.45–5.61
	total	41	1.82	1.06	0.17	1.48–2.15	0.45–5.61

## Discussion

The migration found in this study, as measured by RSA, was of the same magnitude as in other studies of uncemented non-hydroxyapatite coated knee implants (Nilsson et al. 1991, Hilding et al. 1995, Carlsson et al. 2005).

After surgery, the superficial layer of the bone bed is likely to be necrotic and avascular due to the mechanical and thermal trauma of the surgery. In uncemented replacement, the bone bed is usually spared from the high-pressure lavage and from the toxic and thermal trauma of polymethylmetacrylate (Mjöberg 1994). On the other hand, the mechanical loading of the trabeculae underlying the prosthesis will be unevenly distributed, leading to local overload, microfractures and increased sinking, compared to cemented prostheses. Regardless of whether the prosthesis is cemented or not, loss of primary stability will lead to formation of a fibrous membrane, and micromotion will prevent osseous anchorage from being re-established. The membrane thus formed may then allow further osteolysis with time, due to fluid pressure effects (Robertsson et al. 1997, Aspenberg and van der Vis 1998, Van der Vis et al. 1998b) or possibly effects from particles entering the membrane (Horowitz et al. 1991, Kadoya et al. 1997). These secondary phenomena may lead to clinical loosening.

Several authors (Levitz et al. 1995, Regner et al. 1999, Li and Nilsson 2000, Abu-Rajab et al. 2006) have described periprosthetic osteopenia after knee replacement. It could be argued that this bone loss should compromise fixation of the prosthesis. However, this has not been confirmed in studies of revision rate or clinical scoring.

One way of avoiding some of these possibly disadvantageous events is to inhibit bone resorption with bisphosphonates (Wise et al. 2005). A study of clodronate given daily for 6 months postoperatively after knee replacement showed less migration, using RSA, in the treated group (Hilding et al. 2000). All prostheses in that study were implanted with cement and the patients had to take the medication daily for 6 months. Weekly medication with alendronate, as in our study, would simplify the treatment.

In a Cochrane review it was concluded that bisphosphonate treatment reduced periprosthetic bone loss to a higher degree in cemented arthroplasties than in uncemented ones (Bhandari et al. 2005). One possible explanation for this may be that the thermal and toxic trauma from the cement increases bone trauma. This might induce a more intense remodeling reaction than after an operation without cement. The lack of an effect of the bisphosphonate in our study might be explained in several different ways. The study appears not to have been under-powered, as the confidence interval for a difference between the groups was reasonably small. Experiences from a previous study with clodronate and cemented prostheses indicate that 50 patients may be enough to show a decrease by 25% (p = 0.01) (Hilding et al. 2000).

Although we tried to ensure good compliance to the treatment protocol, we cannot be absolutely sure that the patients were actually treated as planned. The medication was not started until the first postoperative day, since we had to know if the fixation had been done without cement before including the patient. This is an obvious difference compared to the study by Hilding et al. (2000), where lower migrations of the tibial components in the bisphosphonate-treated group were found. We had hypothesized that the choice of using uncemented prostheses would be an even better model to test the effects of the bisphosphonates, because uncemented implants migrate more initially, and should therefore have a wider margin for improvement. Our results do not support this theory. One reason for this may be that mechanical fracture of bone trabeculi (rather than bone resorption) causes a large part of the migration of uncemented prostheses, making the advantage of bisphosphonates too small to be measured. There is also the possibility that the treatment dose was too low. We used the dose recommended for treatment of osteoporosis, the dose that has also shown effects in other pathological conditions such as osteonecrosis of the femoral head (Lai et al. 2005). In the study by Hilding et al. (2000), clodronate was used at a dose recommended for treating hypercalcemia and metastatic bone destruction. This can be regarded as a rather high dose. Previous animal studies have indicated that the doses required for inhibition of bone resorption induced by implant instability are higher than the doses needed to affect normal bone remodeling (Åstrand and Aspenberg 2002).

We found no effect regarding migration of the tibial component in uncemented total knee prostheses after 2 years, when using oral alendronate (70 mg weekly) for 6 months starting on the day after surgery. Improvement of the fixation of uncemented prostheses either requires a higher dosage of bisphosphonate, or more attention to surface geometry, more attention to macro and microtexture, or a hydroxyapatite coating.
